# Supplementary material for: A toolbox for surfacing health equity harms and biases in large language models
Source: Nat Med. 2024 Sep 23;30(12):3590–600. doi: 10.1038/s41591-024-03258-2 (PMC11645264; doi:10.1038/s41591-024-03258-2)
Supplement: Supplementary file 2 — Reporting Summary [file 41591_2024_3258_MOESM2_ESM.pdf]

nature portfolio

Corresponding author(s): Stephen Pfohl, Heather Cole-Lewis

Last updated by author(s): Aug 9, 2024

Reporting Summary

Nature Portfolio wishes to improve the reproducibility of the work that we publish. This form provides structure for consistency and transparency in reporting. For further information on Nature Portfolio policies, see our Editorial Policies and the Editorial Policy Checklist.

Statistics

For all statistical analyses, confirm that the following items are present in the figure legend, table legend, main text, or Methods section.

n/a	Confirmed	
☐	☒	The exact sample size (n) for each experimental group/condition, given as a discrete number and unit of measurement
☐	☒	A statement on whether measurements were taken from distinct samples or whether the same sample was measured repeatedly
☐	☒	The statistical test(s) used AND whether they are one- or two-sided<br>*Only common tests should be described solely by name; describe more complex techniques in the Methods section.*
☐	☒	A description of all covariates tested
☐	☒	A description of any assumptions or corrections, such as tests of normality and adjustment for multiple comparisons
☐	☒	A full description of the statistical parameters including central tendency (e.g. means) or other basic estimates (e.g. regression coefficient) AND variation (e.g. standard deviation) or associated estimates of uncertainty (e.g. confidence intervals)
☒	☐	For null hypothesis testing, the test statistic (e.g. F, t, r) with confidence intervals, effect sizes, degrees of freedom and P value noted<br>*Give P values as exact values whenever suitable.*
☒	☐	For Bayesian analysis, information on the choice of priors and Markov chain Monte Carlo settings
☒	☐	For hierarchical and complex designs, identification of the appropriate level for tests and full reporting of outcomes
☒	☐	Estimates of effect sizes (e.g. Cohen's d, Pearson's r), indicating how they were calculated

Our web collection on statistics for biologists contains articles on many of the points above.

Software and code

Policy information about availability of computer code

Data collection

The seven EquityMedQA datasets of adversarial questions were both manually-curated (for which no software is required) and generated semi-synthetically through prompting of the Med-PaLM 2 large language model. The specific prompts used for the latter are included in the supplementary material.

Data analysis

Our data analysis procedure is described in detail in the manuscript. We analyze and visualize data using the Python (version 3.12) and R (version 4.1.3) programming languages. For analysis, we use the statsmodels (version 0.12.2) [1], scipy (version 1.13.1) [2], and krippendorff (version 0.6.1) [3] Python packages. Analytic code to reproduce the primary analyses will be made available at https://github.com/google-research/google-research/health_equity_toolbox.

1. Seabold, S. & Perktold, J. statsmodels: Econometric and statistical modeling with python (2010).
2. Virtanen, P. et al. SciPy 1.0: Fundamental Algorithms for Scientific Computing in Python. Nature Methods 17, 261–272 (2020).
3. Castro, S. Fast Krippendorff: Fast computation of Krippendorff's alpha agreement measure. https://github.com/pln-fing-udelar/fast-krippendorff (2017).

For manuscripts utilizing custom algorithms or software that are central to the research but not yet described in published literature, software must be made available to editors and reviewers. We strongly encourage code deposition in a community repository (e.g. GitHub). See the Nature Portfolio guidelines for submitting code & software for further information.

Data

Policy information about availability of data

All manuscripts must include a data availability statement. This statement should provide the following information, where applicable:

- Accession codes, unique identifiers, or web links for publicly available datasets
- A description of any restrictions on data availability
- For clinical datasets or third party data, please ensure that the statement adheres to our policy

The seven EquityMedQA datasets are available as supplementary data attached to this work (https://doi.org/10.6084/m9.figshare.26133973). The provided data includes the datasets of questions as well as the ratings and generated Med-PaLM 2 and Med-PaLM answers necessary to reproduce the primary analyses of the empirical study. The data includes limited demographic data from the raters (age categories), and does not include the free-text comments from the raters, demographic data for the consumer raters, or the physician-written answers to HealthSearchQA questions.

This work uses the long-form MultiMedQA questions previously described in Singhal et al. [1, 2], which contains samples of questions from HealthSearchQA [1], LiveQA [11], and MedicationQA [12]. This work further uses the set of nine questions studied in Omiye et al. [9].

Research involving human participants, their data, or biological material

Policy information about studies with human participants or human data. See also policy information about sex, gender (identity/presentation), and sexual orientation and race, ethnicity and racism.

Reporting on sex and gender	Participants in the consumer study self-reported their gender from a set of categories defined by the survey vendor.
Reporting on race, ethnicity, or other socially relevant groupings	Participants in the consumer study self-reported their race/ethnicity from a set of categories defined by the survey vendor.
Population characteristics	We report the distribution of the self-reported demographics (age, race/ethnicity, gender) of participants in the consumer study in Supplementary Table 10. To briefly summarize, the population is 18 years of age or older, with ages approximately uniformly distributed across the following categories: 18-24, 25-34, 35-44, 45-54, 55-64, 65+ years of age. The population is 54.7% female and 46.6% male, 57.6% White, 12.6% Black or African American, and 11.1% Hispanic or Latino.
Recruitment	As described in the manuscript, we performed a study with three rater groups: physician, health equity expert, and consumer. The process and criteria used for recruiting physician raters matches that of Singhal et al [1], the qualifications used for recruiting for the health equity expert rater group are described in detail in Extended Data Table 8, and consumer raters were recruited by Qualtrics and partners as part of a set of US-based survey panels sampled based on target age and race/ethnicity distributions representative of the US population.<br>With regards to potential biases attributed to the recruitment process, we note that expert raters (physician and health equity expert raters) were not selected or matched to content to review on the basis of their expertise. As we state in the discussion section, this work covers a large number of identities and contexts, and expert raters may not have expertise appropriate to assess the presence of bias in all cases. Rater experience with related annotation tasks was not assessed nor used as criteria for recruitment. Furthermore, self-selection may induce bias affecting the representativeness of the rater groups.<br>1. Singhal, Karan, et al. "Large language models encode clinical knowledge." Nature 620.7972 (2023): 172-180.
Ethics oversight	This study was conducted under a protocol involving model development and human evaluation using de-identified data, which was reviewed and exempted from further review by Advarra IRB.

Note that full information on the approval of the study protocol must also be provided in the manuscript.

Field-specific reporting

Please select the one below that is the best fit for your research. If you are not sure, read the appropriate sections before making your selection.

☒ Life sciences ☐ Behavioural & social sciences ☐ Ecological, evolutionary & environmental sciences

For a reference copy of the document with all sections, see nature.com/documents/nr-reporting-summary-flat.pdf

Life sciences study design

All studies must disclose on these points even when the disclosure is negative.

Sample size	Formal sample size calculations were not performed. The sizes of the individual datasets of questions introduced in this work are on roughly the same scale (on the order of magnitude of hundreds of questions) as those introduced in prior works e.g., Singhal et al [1,2].<br>1. Singhal, Karan, et al. "Large language models encode clinical knowledge." Nature 620.7972 (2023): 172-180.<br>2. Singhal, Karan, et al. "Towards expert-level medical question answering with large language models." arXiv preprint arXiv:2305.09617

(2023).

Data exclusions	In a small number of cases, raters did not complete the rating task. We remove these instances from the analysis. Further details are provided in the manuscript.
Replication	We replicated analyses with multiple rater groups (physician, health equity expert, consumer) to study heterogeneity on the basis of expertise and perspectives. We further triple-rated a subset of the data for each rater group to support analysis of inter-rater reliability. Heterogeneity within and across rater groups is suggestive of future research directions. No other independent replications of experiments were performed.
Randomization	In analysis of pairwise and counterfactual data (see study methods) the order of examples shown to raters was randomized. Randomization is not relevant to the rest of the study. For example, for analyses involving comparisons across rater groups, every data sample was rated by all the rater groups compared, and thus randomized assignment of the data samples to the rater groups is not relevant.
Blinding	In analysis of pairwise data (see study methods) the order of examples was randomized and the identity of the source of each output was blinded. Blinding is not relevant to the rest of the study, as in no other case did raters evaluate two outputs from different sources side-by-side.

Reporting for specific materials, systems and methods

We require information from authors about some types of materials, experimental systems and methods used in many studies. Here, indicate whether each material, system or method listed is relevant to your study. If you are not sure if a list item applies to your research, read the appropriate section before selecting a response.

Materials & experimental systems

n/a	Involved in the study
☒	☐ Antibodies
☒	☐ Eukaryotic cell lines
☒	☐ Palaeontology and archaeology
☒	☐ Animals and other organisms
☒	☐ Clinical data
☒	☐ Dual use research of concern
☒	☐ Plants

Methods

n/a	Involved in the study
☒	☐ ChIP-seq
☒	☐ Flow cytometry
☒	☐ MRI-based neuroimaging

Plants

Seed stocks	*Report on the source of all seed stocks or other plant material used. If applicable, state the seed stock centre and catalogue number. If plant specimens were collected from the field, describe the collection location, date and sampling procedures.*
Novel plant genotypes	*Describe the methods by which all novel plant genotypes were produced. This includes those generated by transgenic approaches, gene editing, chemical/radiation-based mutagenesis and hybridization. For transgenic lines, describe the transformation method, the number of independent lines analyzed and the generation upon which experiments were performed. For gene-edited lines, describe the editor used, the endogenous sequence targeted for editing, the targeting guide RNA sequence (if applicable) and how the editor was applied.*
Authentication	*Describe any authentication procedures for each seed stock used or novel genotype generated. Describe any experiments used to assess the effect of a mutation and, where applicable, how potential secondary effects (e.g. second site T-DNA insertions, mosiacism, off-target gene editing) were examined.*
